# Insulin-Degrading Enzyme Regulates the Proliferation and Apoptosis of Porcine Skeletal Muscle Stem Cells *via* Myostatin/MYOD Pathway

**DOI:** 10.3389/fcell.2021.685593

**Published:** 2021-10-12

**Authors:** Bingyuan Wang, Jiankang Guo, Mingrui Zhang, Zhiguo Liu, Rong Zhou, Fei Guo, Kui Li, Yulian Mu

**Affiliations:** ^1^Institute of Animal Sciences, Chinese Academy of Agricultural Sciences, Beijing, China; ^2^College of Animal Science and Technology, China Agricultural University, Beijing, China; ^3^Agricultural Genomics Institute at Shenzhen, Chinese Academy of Agricultural Sciences, Shenzhen, China

**Keywords:** insulin-degrading enzyme (IDE), pig, skeletal muscle stem cells, proliferation and apoptosis, myostatin (MSTN)

## Abstract

Identifying the genes relevant for muscle development is pivotal to improve meat production and quality in pigs. Insulin-degrading enzyme (IDE), a thiol zinc-metalloendopeptidase, has been known to regulate the myogenic process of mouse and rat myoblast cell lines, while its myogenic role in pigs remained elusive. Therefore, the current study aimed to identify the effects of IDE on the proliferation and apoptosis of porcine skeletal muscle stem cells (PSMSCs) and underlying molecular mechanism. We found that IDE was widely expressed in porcine tissues, including kidney, lung, spleen, liver, heart, and skeletal muscle. Then, to explore the effects of IDE on the proliferation and apoptosis of PSMSCs, we subjected the cells to siRNA-mediated knockdown of IDE expression, which resulted in promoted cell proliferation and reduced apoptosis. As one of key transcription factors in myogenesis, MYOD, its expression was also decreased with IDE knockdown. To further elucidate the underlying molecular mechanism, RNA sequencing was performed. Among transcripts perturbed by the IDE knockdown after, a downregulated gene myostatin (MSTN) which is known as a negative regulator for muscle growth attracted our interest. Indeed, MSTN knockdown led to similar results as those of the IDE knockdown, with upregulation of cell cycle-related genes, downregulation of MYOD as well as apoptosis-related genes, and enhanced cell proliferation. Taken together, our findings suggest that IDE regulates the proliferation and apoptosis of PSMSCs *via* MSTN/MYOD pathway. Thus, we recruit IDE to the gene family of regulators for porcine skeletal muscle development and propose IDE as an example of gene to prioritize in order to improve pork production.

## Introduction

Skeletal myogenesis is an important and complex process during muscle development, which sequentially involves the proliferation of myoblasts, withdrawal from cell cycle, differentiation into mononucleated myocytes, fusion of myocytes into multinucleated myotubes, and maturation of myotubes into mature muscle fibers (Chal and Pourquié, 2017). Defective muscle development is responsible for several complex human diseases (Huh et al., 2005). Furthermore, in animal species, including pigs and other meat livestock, skeletal muscle provides a source of protein for human nutrition, leading that the extent of muscle development directly affects their commercial value (Jan et al., 2016). Therefore, it is of great importance to illuminate the molecular regulators of skeletal myogenesis, with a long-term aim to improve the treatment of muscle deficiency-related diseases and pig breeding.

Extensive studies have documented that myogenic regulatory factors (MRFs), MYOD (myogenic determination factor), myogenin (MYOG), muscle regulatory factor 5 (Myf5), and muscle regulatory factor 4 (MRF4), coordinately function in different stages of muscle cell fate and play central roles in myogenesis (Asfour et al., 2018). Chronologically, the factors MYOD and Myf5 operate earlier to establish the muscle lineage, by participating in the commitment and proliferation of myogenic-directed cells. Thereafter, MYOG expression further controls the differentiation process, and lastly MRF4, which is involved in myotube maturation (Rawls et al., 1998; Kitzmann and Fernandez, 2001; Yamamoto et al., 2018). Of these four MRFs, MYOD was the first one identified as a myogenic factor since forced expression of MYOD converted fibroblasts to stable myoblasts and activated muscle-specific genes (Davis et al., 1987; Weintraub et al., 1989). Then, MYOG was also identified as a factor regulating myogenesis, since transfection of MYOG into mesenchymal cell line produced cells expressing muscle-specific markers (Wright et al., 1989). Both Myf5 and MRF4 act upstream of MYOD to direct embryonic multipotent cells into the myogenic lineage (Kassar-Duchossoy et al., 2004), demonstrating the importance of MYOD as a downstream effector in myogenesis.

Unlike the downstream effectors, what lies upstream in the myogenic regulatory cascade is less well defined. Among potential candidates, the insulin-degrading enzyme (IDE) was shown to play a relevant role in mouse and rat cell lines. Inhibition of IDE sustained the proliferation of C2C12 myoblasts and blocked the differentiation of C2C12 and L6 myoblasts (Kayalar and Wong, 1989; Epting et al., 2008). IDE is present in humans and in all eukaryotes as well as bacteria, displaying a surprisingly highly conserved primary sequence in all species (Tundo et al., 2017). The biological role of IDE has long been associated with Alzheimer’s disease (AD) and type 2 diabetes mellitus (DM2) due to its well-known substrates amyloid beta-protein (Abeta) and insulin (Duckworth et al., 1998; Kurochkin, 2001). IDE controlled the translocation of insulin to the cell nucleus, playing a crucial role in gene expression and cell proliferation regulated by insulin (Harada et al., 1993). The IDE knockout mice showed increased cerebral accumulation of endogenous Abeta, a hallmark of AD, and had hyperinsulinemia and glucose intolerance, hallmarks of DM2 (Farris et al., 2003). In addition, IDE knockout mice also presented reduced testes weight, reduced seminiferous tubules diameter, and reduced sperm quality (including decreased sperm viability and morphology) compared to wild-type mice (Meneses et al., 2019). These findings indicate the importance of IDE function and bring into its unclear role in porcine skeletal myogenesis.

Therefore, in the present study, we examined the role of IDE in the proliferation of porcine skeletal muscle stem cells (PSMSCs) and the underlying molecular mechanism, aiming to better understand the molecular regulation of porcine muscle development and prioritize candidate factors for the improvement of meat production. Using a knockdown cell line model, we established that albeit IDE was widely expressed in porcine tissues, its downregulation in PSMSCs promoted cell proliferation and counteracted apoptosis *via* myostatin (MSTN)/MYOD pathway.

## Materials and Methods

### Pig Tissue Samples

All animals were treated humanely according to criteria outlined in the “Guide for the Care and Use of Laboratory Animals” published by the Institute of Animal Sciences, Chinese Academy of Agricultural Sciences (Beijing, China). Procedures were approved by the Animal Care and Use Committee (IAS20160616). Pigs (*Sus scrofa*) were slaughtered following the Animal Care Guidelines of the Ethics Committee of Chinese Academy of Agricultural Sciences. Tissue samples including kidney, lung, spleen, liver, heart, and muscle of three 180-day-old male large white pigs (*Sus domesticus*) were collected and kept in liquid nitrogen until further processing.

### Cell Culture and Transfection

Porcine skeletal muscle stem cells were purchased from iCell (iCell-0017a, Shanghai, China). Cells were cultured in high-glucose Dulbecco’s modified Eagle’s medium (DMEM) supplemented with 10% fetal bovine serum (FBS, Gibco), 10 ng/ml human-FGF basic (PEPROTECH), and 1% penicillin streptomycin (Gibco) at 37°C in a humidified incubator containing 5% CO_2_. The siRNAs of IDE (target sequence: GGAATGAAGTTCACAATAA) and MSTN (target sequence: CTCCTAACATTAGCAAAGA) were designed and synthesized by RiboBio (Guangzhou, China). Transfections were performed as previously using Lipofectamine RNAiMAX reagent (Invitrogen) according to the manufacturer’s instructions. Briefly, 1 × 10^5^ cells were seeded in each well of a six-well plate and cultured overnight. Next day, cells were transfected with 50 nM siRNA. After 48 h of transfection, the cells were collected for further analysis.

### Real-Time Quantitative Polymerase Chain Reaction

Total RNA was extracted from pig tissues and PSMSCs using TRIzol reagent (Invitrogen). RNA (1 μg) was reverse transcribed to cDNA using PrimeScript^TM^ RT reagent kit with gDNA eraser (TaKaRa, Cat. # RR047A) according to the manufacturer’s instructions. Real-time quantitative polymerase chain reaction (RT-qPCR) was performed in a final volume of 20 μl which contained 10 μl SYBR^®^ Premix Ex Taq^TM^ (2×; TaKaRa, Cat. # RR420A), 1 μl cDNA, 0.4 μl forward primer (10 μM), 0.4 μl reverse primer (10 μM), 0.4 μl ROX reference dye II, and 7.8 μl sterile distilled H_2_O on an ABI 7500 Fast Real-Time PCR system (Applied Biosystems). Glyceraldehyde-3-phosphate dehydrogenase (GAPDH) was used as a reference gene. Relative mRNA expression was determined by normalizing target gene expression against GAPDH expression and using 2^–ddct^ method (Livak and Schmittgen, 2001). The primer sequences used for RT-qPCR were shown in Supplementary Table 1. Results are presented as fold changes relative to the control.

### Western Blotting Analysis

Total proteins were extracted from pig tissues and PSMSCs using protein extraction reagent containing protease and phosphatase inhibitor (Thermo Scientific). Equal amount of denatured proteins was separated by 10% SDS-PAGE and then transferred to nitrocellulose membranes (Millipore). Thereafter, the membranes were blocked with 5% non-fat milk for 1 h at room temperature followed by primary antibodies incubation overnight at 4°C. The primary antibodies were anti-IDE (1:1000, ab33216) and anti-MSTN (1:1000, ab201954) from Abcam, anti-MYOD (1:500, sc-377460) and anti-MYOG (1:250, sc-13137) from Santa Cruz Biotechnology, and anti-PCNA (1:1000, 2586), anti-CCNE1 (1:1000, 4129), anti-P53 (1:800, 2524), anti-BAX (1:500, 2772), anti-BCL2 (1:500, 3498), and anti-GAPDH (1:1000, 2118) from Cell Signaling Technology. HRP-conjugated secondary antibodies were used to incubate the membranes for 1 h at room temperature. The blots were developed using Pierce ECL Western Blotting Substrate according to the manufacturer’s instructions (Pierce). The protein bands were visualized on a Tanon-5200 Chemiluminescent Imaging System (Shanghai, China) and quantified *via* calculating integrated density with ImageJ software. The protein expression was normalized to endogenous GAPDH.

### Cell Proliferation Assay

The viability of PSMSCs was tested using Cell Counting Kit 8 (CCK-8) (Dojindo Molecular Technologies) according to the manufacturer’s instructions. Briefly, the cells were seeded at a density of 2000 cells per well in 96-well plate and cultured overnight. Then, the cells were treated with siRNAs for 48 h. Thereafter, 10 μl CCK-8 solution was added to each well and incubated at 37°C for 1 h. The absorbance was measured by a microplate reader (Molecular Devices, SpectraMax M5, United States) at the wavelength of 450 nm. The cell viability (%) was calculated with the equation (Absorbance of experimental group − Absorbance of blank)/(Absorbance of control group − Absorbance of blank) × 100. Besides cell viability, the proliferation of PSMSCs was also tested through cell counting. Briefly, the cells were trypsinized after siRNA transfection 48 h, and then counted using a cell counter (JIMBIO CL, China).

### TUNEL Assay for Cell Apoptosis Analysis

The apoptosis of PSMSCs was assayed by TUNEL staining (Beyotime, China) according to the manufacturer’s instructions. The PSMSCs were seeded at a density around 40,000 cells/well in 24-well plate and cultured overnight. The next day, these PSMSCs were transfected with siRNAs. After 48 h of transfection, the cells were fixed with 4% PFA for 30 min at room temperature, which was followed by treatment of Triton X-100 for 5 min. Then, the PSMSCs were treated with TUNEL solution mix for 1 h at 37°C in dark. Last, Hoechst 33342 was used for nuclei staining (Beyotime, China). Each treatment was followed by PBS washing. The TUNEL- and Hoechst-staining PSMSCs were investigated under Leica DMI 6000B microscope and calculated with ImageJ software.

### RNA Sequencing

Total RNA was extracted from PSMSCs (two groups, each with three biological replicates) using TRIzol reagent (Invitrogen). RNA (2 μg) per sample was used for the following steps which were performed by Novogene Co., Ltd. (Beijing, China). Sequencing libraries were generated using NEVNext^®^ Ultra^TM^ RNA Library Prep Kit for Immumina^®^ (NEB, United States) following manufacturer’s instructions. The library quality was assessed on the Agilent Bioanalyzer 2100 system, and the library preparations were sequenced on an Immumina NovaSeq platform and 150 bp paired-end reads were generated.

### RNA Sequencing Data Analysis

Raw data (raw reads) of fastq format were firstly processed. Clean reads were obtained by removing reads containing adapter, reads containing ploy-N, and low quality reads from raw data. Q20, Q30, and GC content of the clean data were calculated. The following analyses were all based on the clean reads with high quality scores. Hisat2 was selected as the mapping tool.

FeatureCounts v1.5.0-p3 was used to count the reads numbers mapped to each gene. And then fragments per kilobase of transcript sequence per millions base pairs sequenced of each gene was calculated based on the length of the gene and reads count mapped to this gene. Differential expression analysis between control groups and treated-groups (two biological replicates per condition) was performed using the DESeq2 R package (1.16.1). The resulting *P*-values were adjusted using the Benjamini and Hochberg’s approach for controlling the false discovery rate. The differentially expressed genes (DEGs) were defined as those genes with an adjusted *P*-value < 0.05 and a | log2(fold change)| ≥ 1.

Gene Ontology (GO) enrichment analysis and Kyoto Encyclopedia of Genes and Genomes (KEGG) enrichment analysis of DEGs were implemented by the clusterProfiler R package, in which gene length bias was corrected. The ENTREZ gene IDs were inputted, and genome-wide annotation for pig was obtained from ftp://ftp.ncbi.nlm.nih.gov/genomes/all/GCF/000/003/025/GCF_000003025.6_Sscrofa11.1. The GO enrichment analysis was performed by the enrichGO function, in which DEGs were divided into three groups: molecular function (MF), cellular component (CC), and biological process (BP). The enrichKEGG function was used for KEGG pathways enrichment analysis. An adjusted *P*-value < 0.05 was considered for significantly enriched GO terms and KEGG pathways.

### Statistical Analysis

Data were analyzed using GraphPad Prism 5 and presented as mean ± standard deviation (SD). The statistical significance was calculated from at least three independent experiments using Student’s two-tailed paired and unpaired *t*-test. *P* < 0.05 was considered significant.

## Results

### The Expression of *IDE* in Pig Tissues

**FIGURE 1:**
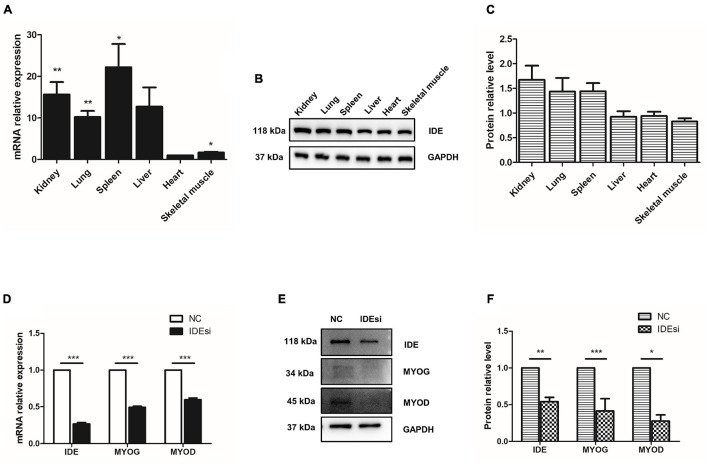
Insulin-degrading enzyme is widely expressed in pig tissues and its knockdown reduces the expression of *MYOD* and *MYOG*. **(A–C)** RT-qPCR assay **(A)** and western blotting analysis **(B,C)** indicate that *IDE* is widely expressed in kidney, lung, spleen, liver, heart, and skeletal muscle of pigs. **(D–F)** *IDE* knockdown reduces the expression of *IDE*, *MYOD*, and *MYOG* at both mRNA **(D)** and protein level **(E,F)** with RT-qPCR assay and western blotting analysis, respectively. *P < 0.05, **P < 0.01, and ***P < 0.001.

In the current study, we aim to explore the role of IDE in PSMSCs. So, firstly, we determined the expression of *IDE* in pig different tissues. mRNA was isolated from kidney, lung, spleen, liver, heart, and skeletal muscle of adult large white pigs and subjected to RT-qPCR. Overall, *IDE* was extensively expressed in these tissues at both mRNA and protein level (Figures 1A–C). In detail, relative mRNA levels of IDE were higher in kidney, lung, spleen, and skeletal muscle, compared to those in heart (Figure 1A). Regarding the protein levels of IDE in these tissues, western blotting analysis showed the protein levels of IDE in kidney, lung, and spleen were slightly higher than those in liver, heart, and skeletal muscle (Figures 1B,C). These findings suggested that *IDE* gene was extensively expressed in multiple tissues of pigs; its expression was regulated to attain different levels in different tissues, suggesting the possible important roles of IDE in pig development.

### *IDE* Knockdown Reduces the Expression of *MYOD* and *MYOG*

During skeletal myogenesis, *MYOD* and *MYOG* as key factors regulate majority of muscle-specific genes and myogenesis process. Thus, to elucidate the possible effect of *IDE* in myogenesis, we tested its impact on the expression of *MYOD* and *MYOG* following *IDE* knockdown using IDE siRNA (IDEsi). The efficacy of *IDE* knockdown was confirmed by RT-qPCR and western blotting. At both mRNA and protein level detection, IDE was significantly reduced after IDEsi treatment (Figures 1D–F). Next, we found that compared to negative control siRNA (NC) group, the expression of *MYOD* and *MYOG* was reduced at both the mRNA (Figure 1D) and protein level (Figures 1E,F) in the *IDE* knockdown group. These results supported that *IDE* might play essential roles in porcine skeletal muscle development.

### Downregulation of *IDE* Promotes the Proliferation of Porcine Skeletal Muscle Stem Cells

**FIGURE 2:**
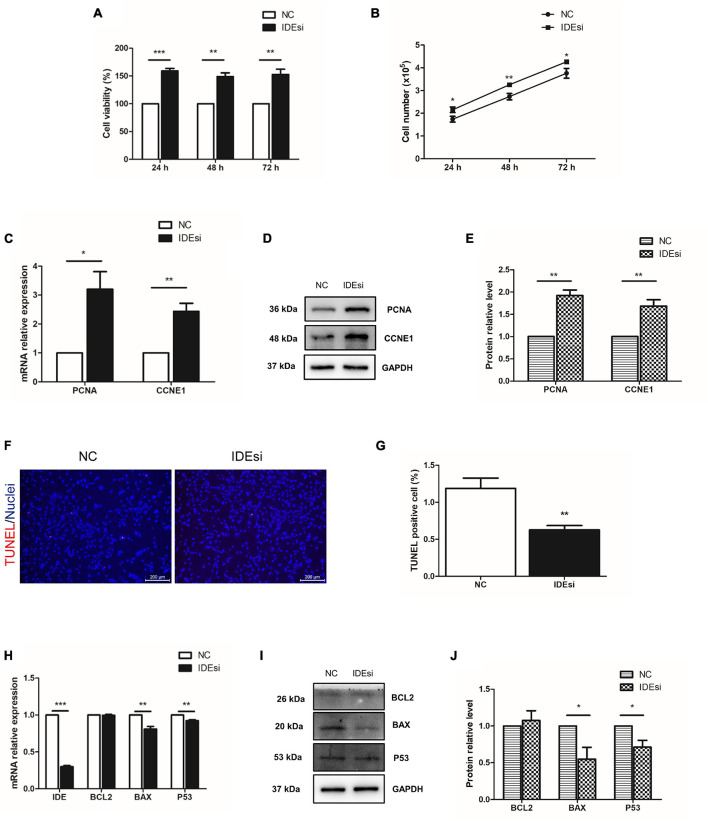
Downregulation of *IDE* promotes the proliferation and mitigates the apoptosis of porcine skeletal muscle stem cells (PSMSCs). **(A)** CCK-8 assay shows that cell viability is increased in IDEsi group than that in NC group after transfection into PSMSCs for 24, 48, and 72 h. **(B)** *IDE* knockdown increases cell number compared with NC group after transfection into the same number of PSMSCs for 24, 48, and 72 h. **(C–E)** mRNA relative expression **(C)** and protein levels **(D,E)** of *PCNA* and *CCNE1* of PSMSCs are increased in IDEsi group than those in NC group *via* RT-qPCR assay and western blotting analysis, respectively. **(F,G)** TUNEL assay discovered that IDE knockdown exhibited less apoptotic cells than control group. **(H)** RT-qPCR assay shows that *IDE* knockdown decreases mRNA relative expression of *IDE*, *BAX*, and *P53*, but has no significant effect on the expression of *BCL2* mRNA. **(I,J)** The protein levels of BAX and P53 but not BCL2 of PSMSCs are declined in IDEsi group than those in NC group. *P < 0.05, **P < 0.01, and ***P < 0.001.

Skeletal muscle stem cells play crucial roles in muscle development and injury-induced muscle regeneration through their proliferation and differentiation. Therefore, building on our above observation that IDE was necessary to sustain the expression of *MYOD* and *MYOG*, we asked whether *IDE* silencing would affect the proliferation of PSMSCs. To this end, we performed cell proliferation analyses using CCK-8 assay and cell number counting. CCK-8 assay revealed that downregulation of *IDE* significantly enhanced cell viability after transfection for 24, 48, and 72 h (*P* < 0.001, *P* < 0.01, and *P* < 0.01, respectively) (Figure 2A). Consistent with the improved cell viability, also the number of PSMSCs was also increased after downregulation of *IDE* as measured after transfection for 24, 48, and 72 h (*P* < 0.05, *P* < 0.01, and *P* < 0.05, respectively) (Figure 2B). The relative expression of cell cycle-related genes *PCNA* and *CCNE1* was increased in IDEsi group than that in NC group, both at the mRNA and protein level (Figures 2C–E). Collectively, these findings indicated that *IDE* negatively regulated the proliferation of PSMSCs.

### *IDE* Inhibition Mitigates the Apoptosis of Porcine Skeletal Muscle Stem Cells

Having identified the negative regulatory role of *IDE* in the proliferation of PSMSCs, we tested whether *IDE* inhibition would mitigate the apoptosis of PSMSCs. To this end, TUNEL assay was performed after siRNA transfection for 48 h. The results showed that compared with control group, IDEsi-treated PSMSCs displayed less TUNEL positive cells (Figures 2F,G). Further, the expression of apoptosis-related genes was examined by RT-qPCR and western blotting. While *IDE* inhibition had no impact on the mRNA expression of *BCL2*, compared to NC group, it significantly decreased the mRNA expression of *BAX* and *P53* (*P* < 0.01) (Figure 2H). Protein levels of BCL2, BAX, and P53 were similar with those of the cognate mRNA (Figures 2I,J), thereby establishing that *IDE* inhibition mitigated the apoptosis of PSMSCs.

### Differentially Expressed Genes Are Screened by RNA Sequencing

**FIGURE 3:**
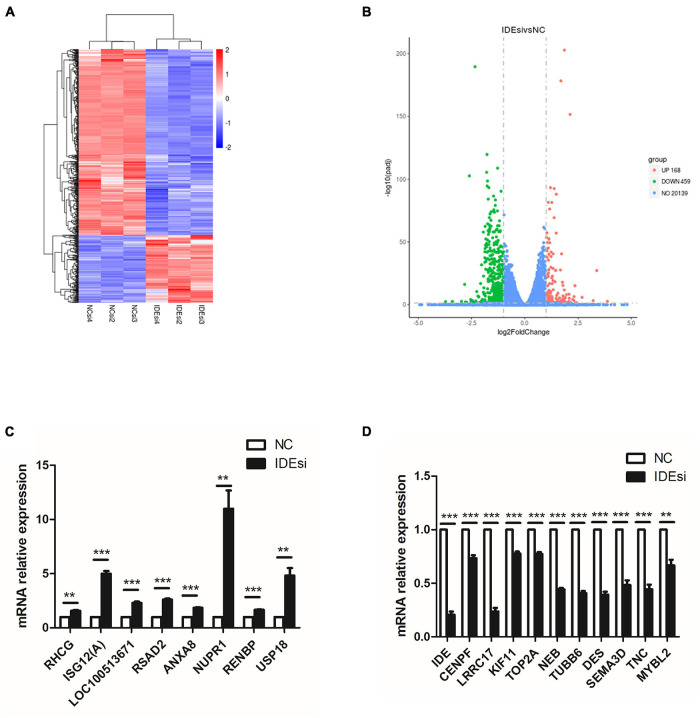
RNA sequencing (RNA-seq) analysis identifies 627 differentially expressed genes (DEGs) with adjusted *P* < 0.05 and | log2 fold change| ≥ 1. **(A)** Heatmap of RNA-seq shows clusters of DEGs in the PSMSCs from IDEsi group and NC group. **(B)** The volcano plot shows 168 upregulated DEGs and 459 downregulated DEGs in IDEsi-transfected PSMSCs compared with NC-transfected PSMSCs. **(C,D)** The mRNA relative expression of randomly selected 8 upregulated DEGs **(C)** and 10 downregulated DEGs **(D)** from RNA-seq are confirmed by RT-qPCR assay. **P < 0.01 and ***P < 0.001.

**FIGURE 4:**
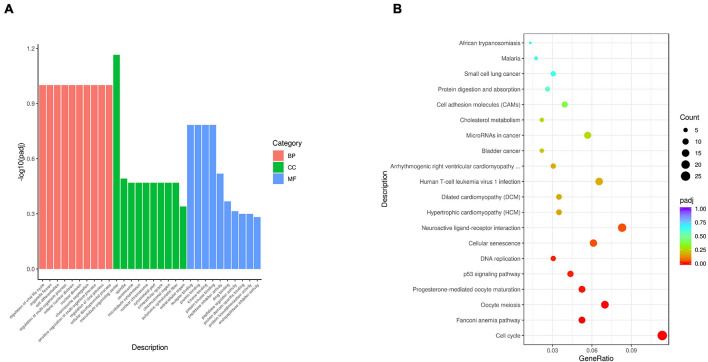
Gene Ontology term enrichment analysis and KEGG pathway analysis are performed with 627 DEGs. **(A)** Representative items for each top 10 terms of biological process (BP), cellular component (CC), and molecular function (MF) are shown in red bars, green bars, and blue bars, respectively. The *x*-axis represents the name of GO terms. The *y*-axis represents –log10 (adjusted *P*-value) of GO terms. **(B)** The top 20 enriched KEGG pathways of 627 DEGs are shown. The *x*-axis represents the gene ratio. The *y*-axis represents the names of KEGG pathways. The gene ratio means the ratio between the number of genes related to the corresponding pathway and the total number of genes in the pathway.

To uncover the molecular mechanism underlying the effect of *IDE* knockdown in PSMSCs, we conducted RNA sequencing (RNA-seq) analysis to compare the transcriptomes between IDEsi-transfected (IDEsi group) and mock-transfected PSMSCs (NC group). A total of 627 mRNAs were differentially expressed (adjusted *P* < 0.05, | log2 fold change| ≥ 1), including 168 upregulated and 459 downregulated differentially expressed genes (DEGs) in IDEsi group compared to NC group (Figures 3A,B). The top 20 upregulated genes which included *IDE* and the top 20 downregulated genes are shown, respectively, in Tables 1, 2, where they are ranked by log2 fold change. To validate RNA-seq results, we subjected a subset of 28 randomly selected DEGs to RT-qPCR. Upregulated DEGs (including *RHCG*, *ISG12(A)*, *LOC100513671*, *RSAD2*, *ANXA8*, *NUPR1*, *RENBP*, *USP18*; Figure 3C) and downregulated DEGs (including *CENPF*, *LRRC17*, *KIF11*, *TOP2A*, *NEB*, *TUBB6*, *DES*, *SEMA3D*, *TNC*, *MYBL2*; Figure 3D) were confirmed. With the comfort of this validation, we undertook a GO analysis of the DEGs and each top 10 terms of BP, CC, and MF were shown in Figure 4A. Further analysis revealed significant enrichment in 11 BP terms from downregulated DEGs (adjusted *P* < 0.05) (Table 3), which included muscle organ development. Further, KEGG enrichment analysis of these DEGs revealed eight significantly enriched pathways, including cell cycle and DNA replication (adjusted *P* < 0.05) (Figure 4B). Thus, the results of DEGs pointed at candidate genes for the molecular mechanism of *IDE* function in PSMSCs.

**TABLE 1:** Top 20 upregulated differentially expressed genes.

**Gene name**	**Gene ID**	**Log2 fold change**	***P* (adj.)**
LOC102165015	102165015	3.870998142	0.002158876
SLC5A7	100512044	3.371005733	5.96E−28
C2CD4C	110259383	3.207169998	0.000587205
MUC12	100286744	2.683796274	1.36E−06
SHC2	110259382	2.435089173	0.023407752
LOC100737730	100737730	2.381840904	0.031178933
HRK	100155596	2.34884663	7.78E−16
SERPINB5	100155836	2.310033525	3.39E−07
TLR4	399541	2.302003156	0.000464508
SCIN	100512981	2.251388026	8.55E−05
PDK4	100286778	2.135906406	0.036212786
LOC106504682	106504682	2.130392455	0.015514156
RHCG	733644	2.121055358	2.67E−152
NKPD1	110261005	2.114110507	0.037505405
SLPI	396886	2.08260702	6.95E−06
LOC110257938	110257938	2.07336763	4.82E−09
CDH8	100625758	2.06017948	0.002443282
WHRN	100520475	2.027474023	0.011737607
LOC110261055	110261055	1.966638858	0.023394409
CASP14	100518472	1.954093983	8.62E−09

*P (adj.) < 0.05 is considered statistical significance.*

**TABLE 2:** Top 20 downregulated differentially expressed genes.

**Gene name**	**Gene ID**	**Log2 fold change**	***P* (adj.)**
KCNA1	100048962	−3.718882073	0.003586753
CPLX1	100624856	−3.410876944	0.003637539
LOC100157763	100157763	−3.396882421	0.003520403
HPGD	100156186	−2.97555016	0.002289409
LYPD5	100626406	−2.827359184	6.63E−17
GALP	396772	−2.789892579	0.007082538
GAL	397465	−2.731537614	0.010545262
XIRP2	397689	−2.602960594	2.44E−103
CLDN9	100302022	−2.541575532	8.67E−05
PTGDR2	100510947	−2.522357235	0.001139225
LOC110260209	110260209	−2.488926485	0.029408964
KCNA3	100156614	−2.454171147	0.000614147
PCSK9	100620501	−2.355200193	0.018361228
IDE	100155309	−2.336970662	2.59E−190
COL13A1	100157199	−2.301938419	0.001092127
MSTN	399534	−2.255207455	3.81E−11
LOC102165115	102165115	−2.230630904	0.030565317
LOC106510075	106510075	−2.198719453	1.35E−07
XKR5	100524909	−2.159402704	7.11E−07
SCN3A	100625056	−2.143679978	0.0291151

*P (adj.) < 0.05 is considered statistical significance.*

**TABLE 3:** Significant enrichment in GO terms.

**Category**	**GO ID**	**Description**	***P* (adj.)**
BP	GO:0000280	Nuclear division	0.022
BP	GO:0007067	Mitotic nuclear division	0.022
BP	GO:0043902	Positive regulation of multi-organism process	0.022
BP	GO:0000278	Mitotic cell cycle	0.022
BP	GO:0048285	Organelle fission	0.022
BP	GO:1903047	Mitotic cell cycle process	0.023
BP	GO:0090068	Positive regulation of cell cycle process	0.027
BP	GO:0010564	Regulation of cell cycle process	0.029
BP	GO:0007517	Muscle organ development	0.037
BP	GO:0030154	Cell differentiation	0.047
BP	GO:0051301	Cell division	0.047

*BP, biological process. *P* (adj.) < 0.05 is considered statistical significance.*

### *IDE* Regulates Porcine Skeletal Muscle Stem Cells *via* Myostatin/*MYOD* Pathway

**FIGURE 5:**
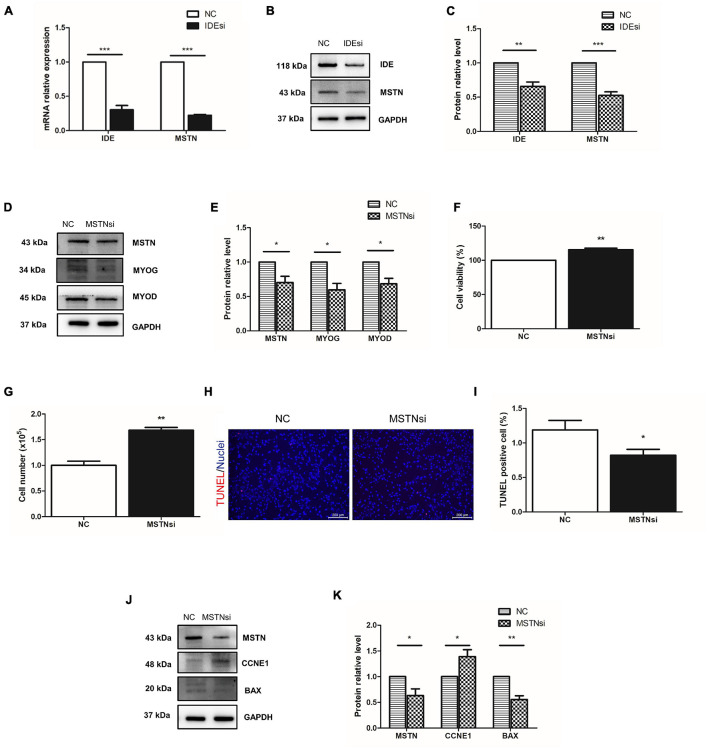
*IDE* regulates PSMSCs through *MSTN*/*MYOD* pathway. **(A)** One of downregulated DEGs, *MSTN*, is confirmed its downregulation in PSMSCs transfected with IDEsi for 48 h compared with that in NC group PSMSCs in both mRNA **(A)** and protein levels **(B,C)** *via* RT-qPCR assay and western blotting analysis, respectively. **(D,E)** *MSTN* knockdown reduces the protein levels of *MSTN* and *MYOD* in PSMSCs. **(F,G)** CCK-8 assay and cell number show that cell proliferation is enhanced in MSTNsi group than that in NC group after siRNA transfection into PSMSCs for 48 h. **(H,I)** TUNEL assay showed that MSTN knockdown exhibited less apoptotic cells than control group. **(G,H,J,K)** Downregulation of *MSTN* leads to increase in the protein level of CCNE1 and decrease in the protein levels of *MSTN* and *BAX* in PSMSCs. *P < 0.05, **P < 0.01, and ***P < 0.001.

Among the DEGs we singled out *MSTN* because it is well known to negatively regulate muscle development and it was downregulated in the IDEsi group compared to NC group (Table 2). To determine whether *IDE* regulated the proliferation of PSMSCs through *MSTN*, we first verified the downregulation of *MSTN* after IDEsi treatment *via* RT-qPCR and western blotting, which exhibited that the expression of *MSTN* was significantly decreased at both the mRNA (Figure 5A) and protein level (Figures 5B,C). Therefore, we subsequently proposed that *IDE* might regulate PSMSCs through *MSTN*. To this end, we explored whether *MSTN* has similar effects with *IDE* in PSMSCs by interfering with the expression of *MSTN* using MSTN siRNA (MSTNsi). Likewise, the protein levels of MYOD and MYOG were reduced with declined MSTN protein level (Figures 5D,E). CCK-8 assay and cell number revealed that cell proliferation was enhanced compared with NC group after MSTNsi treatment (*P* < 0.01) (Figures 5F,G). TUNEL assay showed that compared with control group, MSTNsi-treated PSMSCs exhibited less TUNEL positive number (Figures 5H,I). In addition, *MSTN* knockdown increased the expression of cell cycle-related gene CCNE1 while it decreased the expression of apoptosis-related gene BAX (Figures 5J,K). Thus, these findings uncovered the similar effects of *IDE* and *MSTN* in the proliferation and apoptosis in PSMSCs. Given that *MYOD* functions earlier for establishing the muscle lineage by regulating the commitment and proliferation of myogenic-directed cells and *MYOG* expression controls the differentiation process, our results suggested that *IDE* regulated the proliferation and apoptosis of PSMSCs through *MSTN/MYOD* pathway.

## Discussion

Insulin-degrading enzyme is a neutral zinc and thiol-dependent metallopeptidase and exhibits highly conserved primary sequence in all species (Tundo et al., 2017). Acute exercise augmented IDE expression in skeletal muscle of Swiss mice, implying the potential effect of IDE promoting skeletal muscle development (Kurauti et al., 2016). However, in contrast to clear roles played in AD and DM2 pathologies (Li et al., 2018) and in male reproduction (Meneses et al., 2019), the role of *IDE* remains hypothetical in muscle development, only suggested by studies in mouse and rat myoblast cell lines (Kayalar and Wong, 1989; Epting et al., 2008). Given the commercial value and the importance of pig in agriculture and biomedicine, we designed the present study to test the role of *IDE* in PSMSCs. Our results contribute to a better understanding of the molecular mechanisms of porcine muscle development, and also provide candidate genes for improving pork production.

We first detected the expression of *IDE* in multiple tissues of large white pigs. Although *IDE* was purified from pig skeletal muscle in an earlier study (Yokono et al., 1980), this is the first time that *IDE* expression has been assessed in multiple porcine tissues at once. We found that *IDE* was widely expressed in the tested tissues (kidney, lung, spleen, liver, heart, and skeletal muscle) and exhibited higher expression in kidney, lung, spleen, and skeletal muscle compared to those in heart. Previous studies reported that *IDE* mRNA was highly abundant in kidney and liver of rat (Bondy et al., 1994), and IDE protein was extensively expressed in human tissues, including kidney, liver, lung, brain, and muscle (Yfanti et al., 2008). The similar expression pattern of *IDE* in different species, but at different levels in different tissues, implies conservation of its primary sequence and functional roles.

The development of skeletal muscle is closely related to the health of human and the commercial value of meat livestock, suggesting the importance of myogenesis-related studies. *IDE* was reported as playing importantly regulatory role in mouse myoblasts, since inhibition of *IDE* as well as knockdown of *IDE* mRNA sustained mouse myoblast proliferation (Epting et al., 2008). It is known that myogenesis is a complex process in which the myoblasts proliferate and exit from cell cycle to start differentiation. As the proliferating progeny of satellite cells, the myoblasts express MYOD and Myf5 and undergo multiple rounds of cell division (Le Grand and Rudnicki, 2007). It is known that the proliferation of skeletal muscle stem cells was promoted through keeping MYOD expression at low levels (Conboy and Rando, 2002). MYOD deficiency in satellite cells caused them remaining in proliferative state (Yablonka-Reuveni et al., 1999). MYOD-null myoblasts were more resistant to apoptosis during proliferation (Asakura et al., 2007). In our study, inhibiting *IDE* expression by siRNA transfection impeded the expression of *MYOD*, promoted the proliferation, and attenuated the apoptosis of PSMSCs. Cell proliferation markers *PCNA* and *CCNE1* were also increased after *IDE* inhibition. Therefore, the reduced *MYOD* expression that followed to *IDE* inhibition might contribute to the enhanced proliferation and ameliorated apoptosis of PSMSCs.

To illuminate the molecular bases of *IDE* function in PSMSCs, we applied RNA-seq, comparing IDE knocked-down PSMSCs and control PSMSCs. The RNA-seq data analysis identified 168 significantly upregulated and 459 significantly downregulated genes in the IDE knockdown group compared to control group. As expected, *IDE* was one of the top 20 downregulated genes. Interestingly, we found that *MSTN* was also one of top 20 downregulated genes. *MSTN*, a member of transforming growth factor beta superfamily, has been reported as a negative regulator of muscle growth and development (Steelman et al., 2006; Welle et al., 2007; Gu et al., 2016; Lv et al., 2016; Kim et al., 2020). Numerous studies revealed that loss-of-function mutation of *MSTN* led to double-muscling phenotypes in livestock, including cattle, pig, sheep, and goat (Grobet et al., 1997; Kambadur et al., 1997; Crispo et al., 2015; Qian et al., 2015; Bi et al., 2016; He et al., 2018; Wang et al., 2018), which made *MSTN* a popular candidate gene for animal breeding of improving meat production. *MSTN* operates in muscle development by inhibiting the proliferation and differentiation of myoblast (Thomas et al., 2000; Ge et al., 2020). Thus we posited that *IDE* would regulate the proliferation and apoptosis through *MSTN*. Our knockdown experiment showed that *MSTN* promoted the proliferation of PSMSCs. Furthermore, *MSTN* inhibition also decreased the protein expression of MYOD and BAX, but increased the expression of CCNE1, which exhibited similar results with *IDE* knockdown. Overall, our results suggested that *IDE* possibly regulated the proliferation and apoptosis through *MSTN/MYOD* pathway. Given the report that *IDE* inhibitor treatment suppressed degradation in both subcutaneous and visceral adipocytes (Fawcett et al., 2010), we propose that IDE-loss could also affect fat deposition in pig, which needs to be further explored. The exciting finding of our study is that *IDE* might be a crucial regulator of porcine muscle development and a new candidate for the improvement of pork production. Currently, the limitation could be the unclear role of *IDE* in muscle cell differentiation, the specific functional location of *IDE*, and the possible side-effect of IDE-loss on nervous system and endocrine system. These could be the future direction of work regarding IDE in pig. Nevertheless, with the fast development of genome-editing technology, the specific genetic modified IDE-loss pig could be a valuable breeding material in pig farming and pork industry.

## Data Availability Statement

The datasets presented in this study can be found in online repositories. The names of the repository/repositories and accession number(s) can be found below: NCBI with accessions numbers of samples are SAMN20586768, SAMN20586769, SAMN20586770, SAMN20586771, SAMN20586772, and SAMN20586773.

## Ethics Statement

All procedures conducted in the present study were approved by the Animal Care and Use Committee of Institute of Animal Sciences, Chinese Academy of Agricultural Sciences (ID: IAS20160616).

## Author Contributions

BW and YM conceived and designed the experiments, wrote the manuscript, and obtained the finance supports. JG and MZ mainly performed the experiments. ZL, RZ, and FG prepared the samples and analyzed the data. KL revised the manuscript and obtained the finance supports. All authors read and approved the final manuscript.

## Conflict of Interest

The authors declare that the research was conducted in the absence of any commercial or financial relationships that could be construed as a potential conflict of interest.

## Publisher’s Note

All claims expressed in this article are solely those of the authors and do not necessarily represent those of their affiliated organizations, or those of the publisher, the editors and the reviewers. Any product that may be evaluated in this article, or claim that may be made by its manufacturer, is not guaranteed or endorsed by the publisher.

## Supplementary Material

Supplementary Figure 1 The images of PSMSCs prior and after siRNA transfection (48 h).

Supplementary Table 1 The primers for RT-qPCR used in this study.
