# Supplementary figures and images for: Insulin-Degrading Enzyme Regulates the Proliferation and Apoptosis of Porcine Skeletal Muscle Stem Cells via Myostatin/MYOD Pathway
Source: Front Cell Dev Biol. 2021 Oct 12;9:685593. doi: 10.3389/fcell.2021.685593 (PMC8545900; doi:10.3389/fcell.2021.685593)

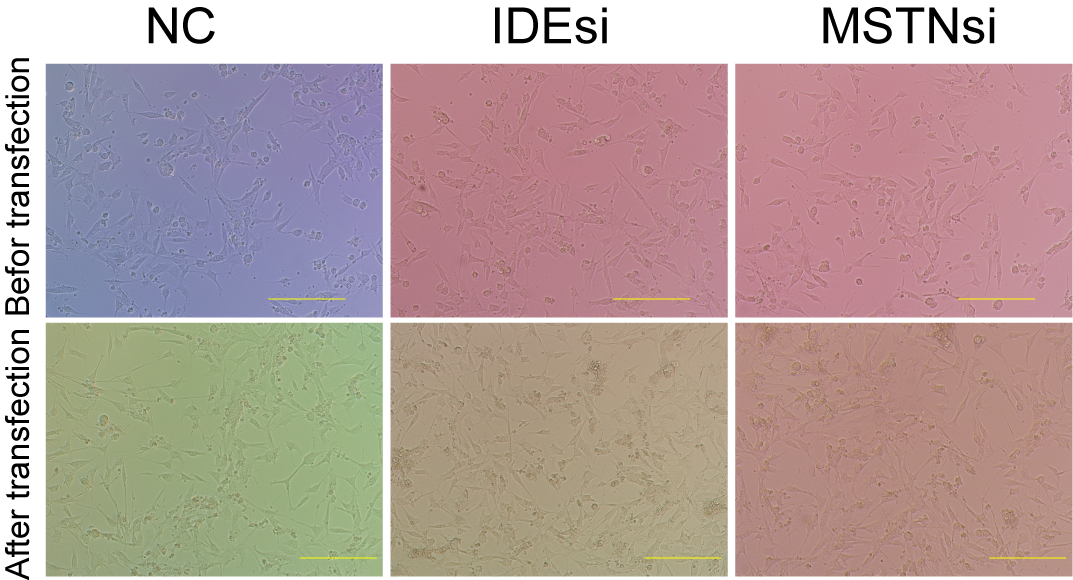

Supplement: Supplementary Figure 1 — The images of PSMSCs prior and after siRNA transfection (48 h). [file Image_1.TIF]
