# Supplementary material for: Insulin-Degrading Enzyme Regulates the Proliferation and Apoptosis of Porcine Skeletal Muscle Stem Cells via Myostatin/MYOD Pathway
Source: Front Cell Dev Biol. 2021 Oct 12;9:685593. doi: 10.3389/fcell.2021.685593 (PMC8545900; doi:10.3389/fcell.2021.685593)
Supplement: Supplementary Table 1 — The primers for RT-qPCR used in this study. [file Table_1.DOCX]

Supplementary Material

# Supplementary Table

**Table S1** The primers for RT-qPCR used in this study

| Gene name |  | Primer sequences |
| --- | --- | --- |
| *GAPDH* | F | AGGGCTGCTTTTAACTCTGGCAA |
|  | R | GATGGTGATGGCCTTTCCATTG |
| *IDE* | F | AAGCAGGCCGCATTAGGAAT |
|  | R | GCTCTGCATGTCTGTCTGGT |
| *MYOD* | F | TGTTCCGACGGCATGATGGAT |
|  | R | TGGGCGCCTCGCTGTAATAG |
| *PCNA* | F | TGCAGATGTACCCCTTGTTGT |
|  | R | TATGTGCTGGCATCACCGAA |
| *CCNE* | F | GGGTCTGGCGAGGTGTCA |
|  | R | ACAGGTGGCCAACAATTCCT |
| *BCL2* | F | GAGGCTGGGATGCCTTTGTG |
|  | R | CCACAGCTTCTTTGCATGGT |
| *BAX* | F | GCCCTTTTGCTTCAGGGTTTC |
|  | R | TGCCGTCAGCAAACATTTCG |
| *P53* | F | TGACTGTACCACCATCCACTAC |
|  | R | AAACACGCACCTCAAAGC |
| *RHCG* | F | GCCTCATCGTAGGGGTCATT |
|  | R | GGCAGAGGAGGTGGAAACTC |
| *ISG12(A)* | F | AGCTCCGTGGTTGCAGAAAA |
|  | R | AGACTCCCTGTCGCCAGTAT |
| *LOC100513671* | F | TGTTGTCGTCACAGGATGGG |
|  | R | CCCTGGCAGGCATCGATATT |
| *RSAD2* | F | AAAGCTCTGAACCCTGTCCG |
|  | R | CTTCCGCCCGTTTCTACAGT |
| *ANXA8* | F | CTTCTGAGTGCAGCAGGGG |
|  | R | CCGATCCCCTTCATGGCTTT |
| *NUPR1* | F | GGAAAGGTCGCACCAAGAGA |
|  | R | GTCACGTGGGGTAAGTCCTG |
| *RENBP* | F | GAGCTTCTGGATGCGGCTAA |
|  | R | GGCCATGGTGTAGAAGCACT |
| *USP18* | F | TACCTCACCGTCTGGAACCT |
|  | R | CTGTTCCTGACATGCTCCGT |

Continued Table S1

| Gene name |  | Primer sequences |
| --- | --- | --- |
| *CENPF* | F | TTGCAACGAGACCCTCAGTC |
|  | R | AGCCGTTCAGCTCGGATATG |
| *LRRC17* | F | TCAGGCCCAACAACACAACT |
|  | R | AGATGAGGGCAAGGCTAGGA |
| *KIF11* | F | AAACTGGCACCGGAAAAACG |
|  | R | TCCCCTCTTGTTACGGGGAT |
| *TOP2A* | F | GGTGCTGATAAATGGCGCTG |
|  | R | TCTGATGGGAAGCTCGGAGA |
| *NEB* | F | ACAAGGGTTGCTTCACACCA |
|  | R | ACTGAGCCAGGGTTTGTACG |
| *TUBB6* | F | GGGCCAGTGCGGGAATC |
|  | R | CACCAGTCTGTCCGAAGATGAA |
| *DES* | F | GATCCAGTCCTACACCTGCG |
|  | R | GATAGGGAGGTTGATCCGGC |
| *SEMA3D* | F | ATCTGTTGGCTGAGTCACGG |
|  | R | CATGTGCTTCCACTTTGGGC |
| *TNC* | F | AGGTTCCTGGAGACGATGGA |
|  | R | TCTGGGGTGGCATCTGAAAC |
| *MYBL2* | F | AGAGCAGCGAGACAGCAAAT |
|  | R | TGCTTCGTGCCGTACTTCTT |
| *MSTN* | F | ACCCAGGCACTGGTATTTGG |
|  | R | GGTCCTGGGAAGGTTACAGC |
